# The ameliorative role of specific probiotic combinations on bone loss in the ovariectomized rat model

**DOI:** 10.1186/s12906-022-03713-y

**Published:** 2022-09-17

**Authors:** Ahmad Gholami, Mohammad Hossein Dabbaghmanesh, Younes Ghasemi, Farhad Koohpeyma, Pedram Talezadeh, Nima Montazeri-Najafabady

**Affiliations:** 1 Biotechnology Research Center, Shiraz University of Medical Sciences, Shiraz, Iran; 2 Pharmaceutical Science Research Center and School of Pharmacy Shiraz University of Medical Sciences, Shiraz, Iran; 3 Endocrinology and Metabolism Research Center, Nemazee Hospital, Shiraz University of Medical Sciences, Shiraz, Iran

**Keywords:** Probiotic, Osteoporosis, Lactobacillus, Bone

## Abstract

**Background:**

Osteoporosis, a skeletal disease described by impaired bone strength, cause an increased risk of fractures. We aimed in this study to clarify which particular wise combination of probiotics has the most beneficial effect in the rat model of osteoporosis.

**Methods:**

Sixty-three mature female Sprague Dawley rats (12–14 weeks old, weight 200 ± 20 g) were ovariectomized and then divided into nine random groups, each group consisting of 7 rats. Lactic acid bacteria were isolated from traditional fermented yogurt on the northern coast of the Persian Gulf. Seven combinations of probiotics, each containing three probiotic strains, were designed and administered (1 × 10 ^9^ CFU / ml/strain daily along with their water) to treat ovariectomized rats. The period from ovariectomy to eutanásia was 3 months. For evaluating femur, spine, and tibia, bone mineral density (BMD), and bone mineral content (BMC), Dual-energy X-ray absorptiometry (DEXA) scans were performed. Also, effect of probiotic combinations was assessed on biochemical markers including vitamin D, calcium, phosphorus, and alkaline phosphatase in serum.

**Results:**

Combination NO 4, containing *L. acidophilus, B. longum,* and *L. reuteri,* is the most influential group on global, spine, and femur BMD. Combination NO 3, containing *L. acidophilus*, *L. casei*, and *L. reuteri,* also significantly affects the BMD of the tibia among the treatment group. We found that the combination NO 4 had the most significant ameliorative effect on global BMC. Also, combination NO 1 (comprising *L. acidophilus, L. casei,* and *B. longum)*, NO 6 (containing *L. casei, B. longum,* and *Bacillus coagulans),* NO 7 (containing *L. casei, L. reuteri,* and *B. longum),* and NO 4 had the most considerable raising effect on spine BMC. In addition, the serum calcium and Vitamin D concentration in the groups NO 4, 6, and 7 were significantly higher than in OVX groups, whereas the alkaline phosphatase concentration was considerably reduced in these groups.

**Conclusion:**

Among nine effective probiotics, a combination containing *L. acidophilus, B. longum,* and *L. reuteri* is the most influential group in ovariectomized osteoporotic rat.

## Introduction

Osteoporosis, a skeletal disease described by impaired bone strength that causes an increased risk of fractures, is a worldwide disease that mainly occurs in postmenopausal women and older adults [1, 2]. The condition is a bothering factor for about 200 million people, with low bone mass, worsening of the fine structure of bone tissue, and elevated chance of fractures [3]. Since the population is getting old, it is predictable that the prevalence of osteoporosis will significantly increase [4].

Probiotics are living microorganisms that bring health benefits to consumers by promoting intestinal balance [5, 6]. They contain different microbes explained by their genus, species, and strain designation [7]. Probiotic supplements are beneficial for humans and animals and can be used after antibiotic therapy to compensate for gut microbiome deficiency or as prophylaxis [8]. The International Scientific Association of Probiotics and Prebiotics (ISAPP) has a helpful definition of probiotics consisting of three properties; probiotics should be alive during administration, be healthy for users, and have the right dose when delivered [9].

Many experiments have been recently done to specify the potential profits of probiotic preparations in pathologic bone disorders such as osteoporosis. In this regard, several probiotic strains have been introduced in a single administration or as food supplementation to ameliorate bone loss [2]. Oral intake of a single probiotic strain *Limosilactobacillus reuteri* (*L. reuteri*) for 28 days by male rat caused substantial health benefits for trabecular bone density, number, thickness, and bone mineral content and density both in the femur and vertebral [10]. A study showed that when a single strain of *Bifidobacterium longum* (*B. Longum*) was orally administered to male rats for 4 weeks, the microelements essential for bone health, such as calcium, phosphorus, and magnesium, were concentrated in the tibia [11]. Also, *Lacticaseibacillus paracasei* (*L. paracasei*) could increase cortical bone mineral content, and the resorption marker C-terminal telopeptides in rat blood were decreased as well as calcium exertion through urine [12]. This probiotic strain stimulates differentiation of osteoblasts via bone morphogenetic protein (BMP) and inhibits RANKL-induced differentiation of osteoclast, thus helping to inhibit bone loss. Dar et al. reported that *Lactobacillus acidophilus* (*L. acidophilus*) improves the microarchitecture of both trabecular bone and cortical bone, enhancing bone mineral density and heterogeneity immunomodulatory impact on the host immune system [13]. Also, we observed that the supernatant of *Bacillus coagulans* (*B. coagulans*) enriched the tibia bone mineral density (BMD) of OVX rat in the previous study [2]. In addition, our previous studies evaluated the effects of isolated several native strains of probiotics on bone loss in ovariectomized rats [3]. Although their mechanism of action hasn’t been entirely understood, these probiotics can affect the regulation of luminal pH, production of enzymes, organic acids, and antimicrobial peptides, improvement of barrier function by enriching mucus secretion, stimulation of osteoblast differentiation, and maintaining of the host immune system [2, 14]. They also may have effects on preventing and treating osteoporosis by affecting calcium absorption via decreasing pH, inhibiting calcium binding to bile acid and elevating the surface area for absorption in the large intestine, regulating the immune responses, and producing small molecules, for instance, serotonin or estrogen-like molecules [15–17].

Combining several probiotic strains appears to have greater efficacy against many diseases than single-strain therapy. Few studies have shown that multi-strain probiotics, including the strains that are a component of the combination itself, are more potent [18]. However, it is still unclear whether this superiority is because of synergistic effects between the strains or because of the high dose of probiotics used.

To accurately demonstrate the greater efficacy of multivariate probiotics and which multivariate probiotic compound is more effective for treating osteoporosis, further studies using the same doses in similar populations are needed. Given the positive effect of probiotics alone or in randomized combination in the treatment of osteoporosis, we aimed in this study to clarify which particular wise combination of probiotics has the most beneficial effect in the postmenopausal rat model. Therefore, due to the positive impact of probiotics alone in the treatment of osteoporosis, we configured them in seven selective combinations and compared their effectiveness in protecting rats from ovariectomized (OVX)-induced bone loss. As there are several probiotic combinations in the supplements market, this study aimed to determine which probiotic combination is more effective in ameliorating bone loss in OVX rats using quantitative indicators.

## Material and methods

### Isolation and formulation of bacteria

Twenty samples of traditional fermented yogurt from the northern coast of the Persian Gulf were used for lactic acid bacterial isolation. After preparation, the samples were stored in a refrigerator at 4 °C. 10 g of each was diluted in peptone water (4%), homogenized with a laboratory mixer, and serially diluted with sterile water. To count the *Lactobacillus*, *Streptococcus,* and *Bifidobacterium*, LS differential medium was used. De Man, Rogosa, Sharpe (MRS) agar, and *Bifidobacterium* medium (BFM) agar isolate *Lactobacilli* and *Bifidobacteria*. All plates were incubated under anaerobic conditions at 37° C for three days. According to Bergey's Manual of Systematic Bacteriology, MRS and BFM agar isolates were identified based on cultural, morphological, and biochemical properties. After identification, the strain was maintained by subculture in tryptic soybean agar (TSA) medium at 37° C and stored in tryptic soybean broth (TSB) medium at 4° C until lyophilization (maximum storage days was two weeks). Before use, the lyophilized strain was formulated with phosphate-buffered saline (PBS, pH 7.4) and mechanically stirred for 15 min to mix well. The probiotic PBS solution premix was prepared for oral tube feeding. The concentration of probiotic candidates at each interval was 1.5 × 10^8^ colony forming units (CFU) / ml.

An equal amount of each strain mentioned in Table 1 was used to prepare the probiotic combinations to reach the final concentration of 10^9^. To ensure that our combination samples were qualified and had no contaminant, every sample given to the animals was cultured.

**Table 1:** Content of specific combinations of probiotics used in this study

1	*Lactobacillus acidophilus*	*Lacticaseibacillus casei*	*Bifidobacterium longum*
2	*Lactobacillus acidophilus*	*Lacticaseibacillus casei*	*Bacillus coagulans*
3	*Lactobacillus acidophilus*	*Lacticaseibacillus casei*	*Limosilactobacillus reuteri*
4	*Lactobacillus acidophilus*	*Bifidobacterium longum*	*Limosilactobacillus reuteri*
5	*Lactobacillus acidophilus*	*Bifidobacterium longum*	*Bacillus coagulans*
6	*Lacticaseibacillus casei*	*Bifidobacterium longum*	*Bacillus coagulans*
7	*Lacticaseibacillus casei*	*Limosilactobacillus reuteri*	*Bifidobacterium longum*

### Experimental design

Sixty-three adult female Sprague Dawley rats (12–14 weeks old, weight 200 ± 20 g) were purchased from the Laboratory Animal Center of Shiraz Medical University. Rats were brought up under typical laboratory conditions (room temperature (23 ± 2° C), 60 ± 5% relative humidity, and 12/12-h light / dark cycle) with a standard pellet diet and water and given freely. A standard pellet diet composition was as follows: Crude protein 23%, crude fat 3.5%, crude fiber 4.5%, ash 10%, calcium 0.95–1%, phosphorus 0.65–0.7%, NaCl 0.5%, lysine 1.15%, methionine 0.33%, threonine 0.72%, tryptophan 0.25%, cysteine 0.3%). Rats were acclimatized to the animal room for one week. They were then divided into nine random groups, each consisting of 7 rats. The classification of animals is according to this:

- group 1, control;
- group 2, OVX;
- group 3, OVX + combination NO 1;
- group 4, OVX + combination NO 2;
- group 5, OVX + combination NO 3;
- group 6, OVX + combination NO 4;
- group 7, OVX + combination NO 5,
- group 8, OVX + combination NO 6,
- group 9, OVX + combination NO 7.

The contents of each combination are mentioned in Table 1.

The animals in groups 3, 4, 5, 6, 7, 8, and 9 were treated with 1 ml (1 × 10 ^9^ CFU / ml/strain daily along with their water) of probiotics for four weeks. Normal saline was supplied to the rats of groups 1 and 2. Food and water absorption were monitored and did not differ between groups. This work was approved by the Ethics Committee (NO. IR.SUMS.REC.1398.500) of Shiraz University of Medical Sciences, Shiraz, Iran.

### Ovarectomy procedure

Adult female rats were bilaterally ovariectomized. The period of ovariectomy until euthanasia was 3 months. Ketamine 10% (100 mg/kg, Alfasan, Netherlands) and xylazine 2% (10 mg/kg, Alfasan, Netherlands) were the anesthetics used in this study. Both ovaries were resected in all groups, except for the first group, which was the control group, after surgical anastomosis of the uterine horns through a central longitudinal incision. Sham operation was performed in the control group.

### Dual-energy X-ray absorptiometry parameter measurements

In order to assess the area, bone mineral content (BMC), and bone mineral density (BMD) of femur, spine, and tibia, Dual-energy X-ray absorptiometry (DXA) scans were applied on a Discovery QDR, USA device with Hologic instrument via the particular software for small animals at the experiment termination. At first, we set up the RAT STEP PHANTOM (Hologic P/N010-0758Rev.004) scan. In this method, when the system motion was completed, we centered the STEP PHANTOM on the table along the long axis of the laser with the cross-hair ¾ "(2 cm) of the right edge of the thinnest step. Then we pressed a continue button to start the scan. BMC in grams, bone area (BA) in square centimeters, and BMD in g/cm2 were measured.

### Biochemical analysis of serum

Blood samples were collected in chilled non-heparinized tubes for clotting at room temperature by cardiocentesis. Then they were centrifuged at 3500 rpm at 4 ͦC for 20 min, and biochemical markers including vitamin D, calcium (Ca), phosphorus (P), and alkaline phosphatase (ALP) were measured by assessing isolated sera (Fig. 3).

### Statistical analysis

Statistical analysis was performed using IBM © SPSS © Statistics v 22.0 for Windows. The data are expressed as mean ± standard deviation. One-way analysis of variance (ANOVA) was applied to analyze the association between existing probiotic combinations and bone mineral density measurement parameters. Tukey post hoc analysis was performed when the ANOVA outcomes exhibited significance (*p* ≤ 0.05).

## Results

### Bacterial strains isolation and identification

The physiological and biochemical properties of the five bacterial strains applied in this study are mentioned individually in Table 2. The isolated probiotics are *Lactobacillus acidophilus, Limosilactobacillus reuteri, Lacticaseibacillus casei, Bifidobacterium longum,* and *Bacillus coagulans (*previously known as *Lactobacillus sporogenes)* based on the standard references and morphological characteristics.

**Table 2:** The physiological and biochemical properties of the selected strains

Type of test	*B. coagulase*	*L. acidophilus*	*B. longum*	*L. reuteri*	*L. casei*
Growth at 15 ͦC	+	-	-	+	+
Growth at 45 ͦC	+	-	-	+	+
VP	+	-	-	+	-
Nitrate reduction	-	-	-	-	-
Gas production from glucose	-	-	-	+	+
Resistance to bile salts	+	+	+	+	+
Motility	+	-	-	-	-
Catalyze	+	-	-	-	-
Oxidase	-	-	-		
Arabinose	+	+	+	+	-
Inositol	-	-	-	-	-
Inulin	-	-	-	-	-
Raffinose	-	+	+	-	-
Rhamnose	-	-	-	-	-
Cellobiose	+	+	-	-	+
Sorbose	-	-	-	-	-
Glucose	+	+	+	+	+
Sorbitol	-	+	-	-	+
Fructose	+	+	+	-	+
Galactose	+	-	+	-	+
Cellulose	-	-	-	+	-
Lactose	-	-	+	+	+
Mannose	+	+	-	-	+
Mannitol	+	+	-	-	+
Melazitose	+	+	-	-	+
Melibiose	-	-	+	+	+
Maltose	+	+	+	+	+
Ribose	+	+	+	+	+
Sucrose	+	+	+	+	+
Trehalose	-	+	-	-	+
Xylose	-	-	+	+	-

### Effect of probiotic combinations on bone mineral density

**Fig. 1:**
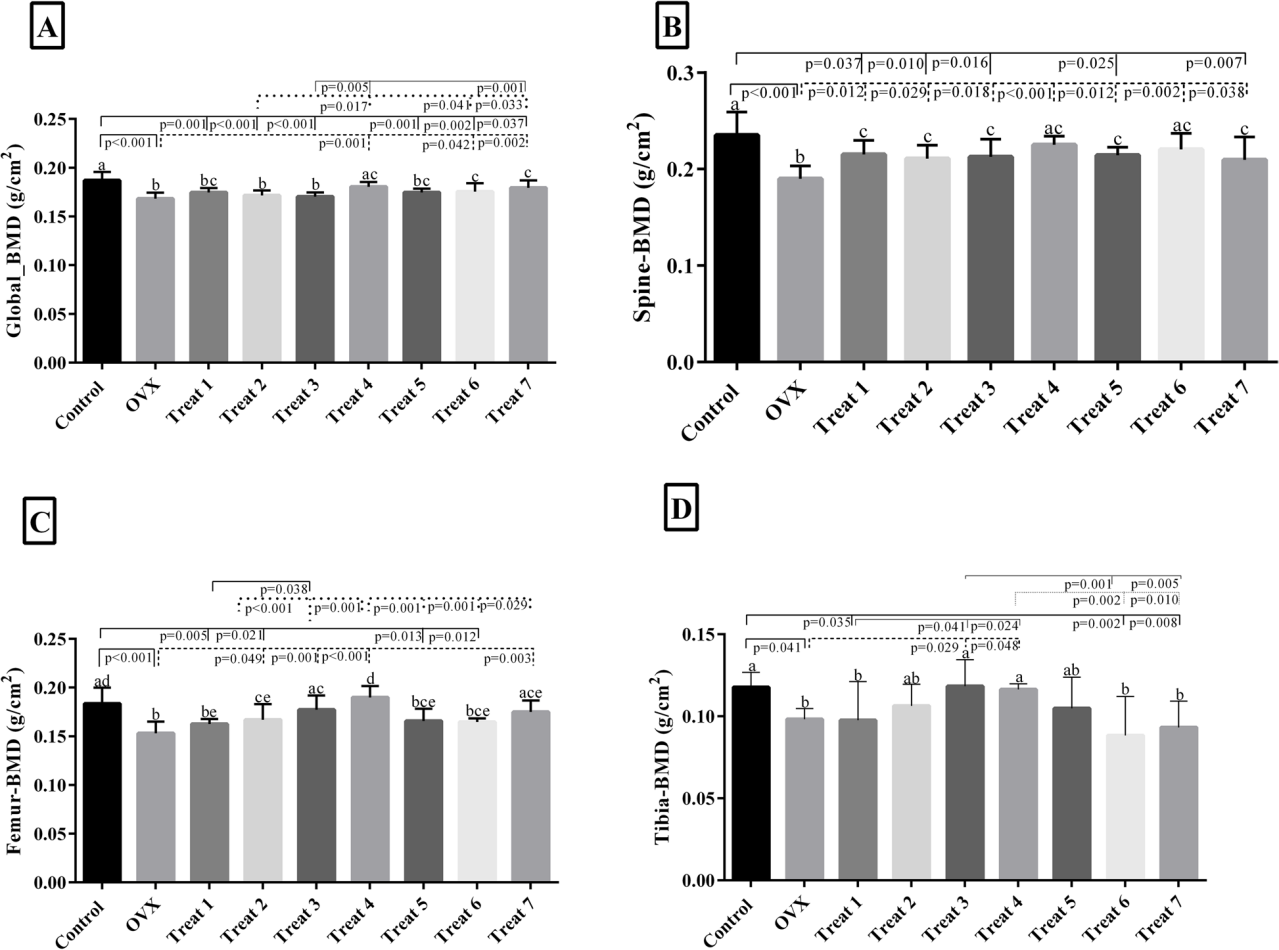
The effect of the probiotic combinations on the overall spine, femur, and tibia BMD. Treat 1: OVX + combination of *Lactobacillus acidophilus*, *Lacticaseibacillus casei*, *Bifidobacterium longum*; Treat 2: OVX + combination of *Lactobacillus acidophilus*, *Lacticaseibacillus casei*, *Bacillus coagulans*, Treat 3: OVX + combination of *Lactobacillus acidophilus*, *Lacticaseibacillus casei*, *Limosilactobacillus reuteri*; Treat 4: OVX + combination of *Lactobacillus acidophilus, Bifidobacterium longum*, *Limosilactobacillus reuteri*; Treat 5: OVX + combination of *Lactobacillus acidophilus*, *Bifidobacterium longum*, *Bacillus coagulans*; Treat 6: OVX + combination of *Lacticaseibacillus casei*, *Bifidobacterium longum*,*Bacillus coagulans*; Treat 7: OVX + combination of *Lacticaseibacillus casei*, *Limosilactobacillus reuteri*, *Bifidobacterium longum*. There were no significant differences between columns, which have at least one similarly letters. However, dissimilar letters indicate significance (*P* < 0.05)

Figure 1 displays the effect of the probiotic combination on the overall spine, femur, and tibia BMD. Combinations # 4, 6, and 7 significantly ameliorated the global BMD of the OVX-treated group compared to the untreated OVX group. Combinations NO 1, 2, 3, and 5 had no significant increasing effect on the global BMD (Fig. 1a). Spine BMD of treated OVX groups was significantly increased when treated with all of the probiotic combinations vs. untreated OVX group (Fig. 1b). Combinations NO 2, 3, 4, and 7 increased the femur BMD remarkably in the treated OVX groups in comparison with the untreated group. Combination NO 1, 5, and 6 had no considerable effect on treated groups (Fig. 1c). In terms of tibia BMD, combinations NO 3 and 4 considerably increased BMD vs. untreated OVX group. In contrast, combinations NO 6 and 7 had a minor effect on BMD (Fig. 1d).

### Effect of probiotic combinations on bone mineral content (BMC)

**Fig. 2:**
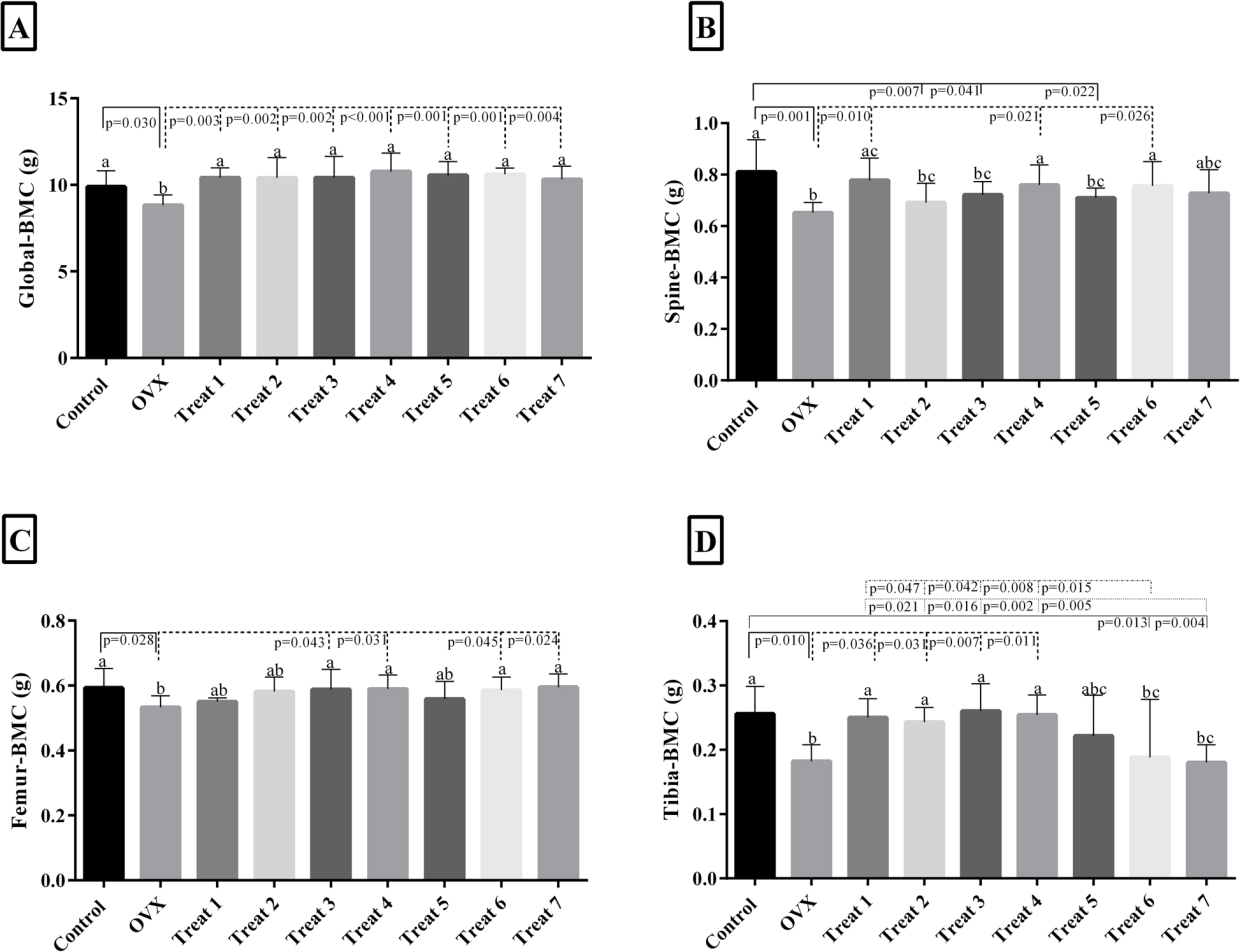
The effect of a probiotic combinations on global, spine, femur, and tibia BMC. Treat 1: OVX + combination of *Lactobacillus acidophilus*, *Lacticaseibacillus casei*, *Bifidobacterium longum*; Treat 2: OVX + combination of *Lactobacillus acidophilus*, *Lacticaseibacillus casei*, *Bacillus coagulans*, Treat 3: OVX + combination of *Lactobacillus acidophilus*, *Lacticaseibacillus casei*, *Limosilactobacillus reuteri*; Treat 4: OVX + combination of *Lactobacillus acidophilus, Bifidobacterium longum*, *Limosilactobacillus reuteri*; Treat 5: OVX + combination of *Lactobacillus acidophilus*, *Bifidobacterium longum*, *Bacillus coagulans*; Treat 6: OVX + combination of *Lacticaseibacillus casei*, *Bifidobacterium longum*,*Bacillus coagulans*; Treat 7: OVX + combination of *Lacticaseibacillus casei*, *Limosilactobacillus reuteri*, *Bifidobacterium longum*. There were no significant differences between columns, which have at least one similarly letters. However, dissimilar letters indicate significance (*P* < 0.05)

Figure 2 shows the effect of a probiotic combination on global, spine, femur, and tibia bone mineral content (BMC). As exists in Fig. 2a, all combinations significantly augmented the global BMC of OVX groups compared to untreated. Spine BMC in groups treated with combinations NO 1, 4, and 6 have significant differences from the untreated group (Fig. 2b). In the femur, the combinations NO 3, 4, 6, and 7 significantly increased the BMC (Fig. 2c). The BMC of the tibia was considerably increased while treated with combinations NO 1 to 4 and had no substantial change when treated with combinations NO 6 and 7 (Fig. 2d).

### Effect of probiotic combinations on biochemical factors in serum

**Fig. 3:**
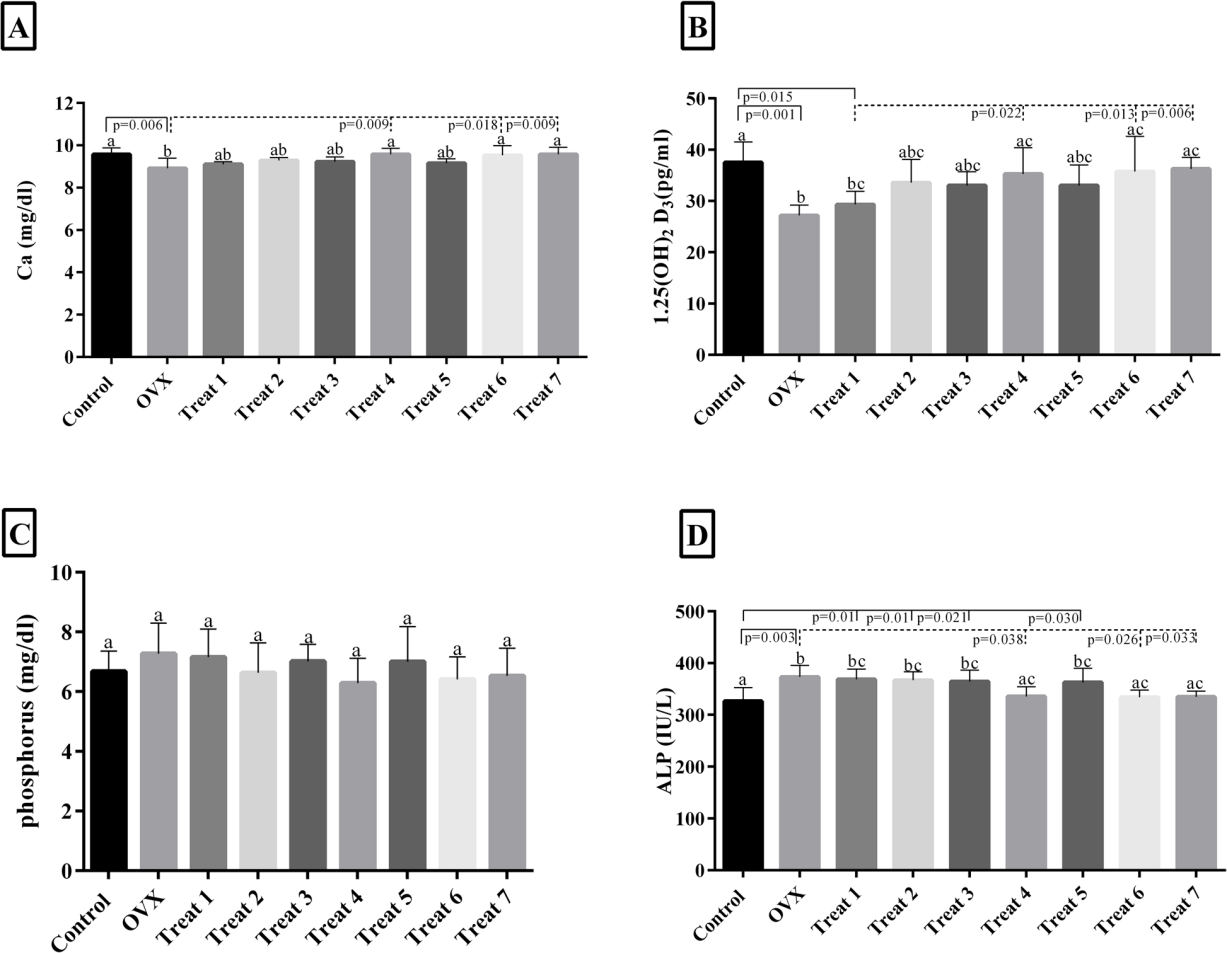
The effect of probiotics on serum calcium (**A**), 1, 25 (OH)2 vitamin D (**B**), phosphorus (**C**), and alkaline phosphatase (**D**) concentrations in ovariectomized rats. Treat 1: OVX + combination of *Lactobacillus acidophilus*, *Lacticaseibacillus casei*, *Bifidobacterium longum*; Treat 2: OVX + combination of *Lactobacillus acidophilus*, *Lacticaseibacillus casei*, *Bacillus coagulans*, Treat 3: OVX + combination of *Lactobacillus acidophilus*, *Lacticaseibacillus casei*, *Limosilactobacillus reuteri*; Treat 4: OVX + combination of *Lactobacillus acidophilus, Bifidobacterium longum*, *Limosilactobacillus reuteri*; Treat 5: OVX + combination of *Lactobacillus acidophilus*, *Bifidobacterium longum*, *Bacillus coagulans*; Treat 6: OVX + combination of *Lacticaseibacillus casei*, *Bifidobacterium longum*,*Bacillus coagulans*; Treat 7: OVX + combination of *Lacticaseibacillus casei*, *Limosilactobacillus reuteri*, *Bifidobacterium longum*. There were no significant differences between columns, which have at least one similarly letters. However, dissimilar letters indicate significance (*P* < 0.05)

As shown in Fig. 3A, serum Ca concentration was significantly lower in the OVX group compared to the control group at the end of the experiment (*P* ≤ 0.006). In treated groups no. 4, 6, and 7, the Ca concentration was significantly higher compared to OVX group (Fig. 3A). These differences between group 4,6,7 and the control group were insignificant (*P* > 0.05). The concentration of Ca was significantly (*P* ≤ 0.05) lower in groups no. 1,2,3 and 5 compared to the control group, although they significantly increased Ca level compared to the OVX group (Fig. 3A).

As expected, the serum VitD concentration was lower in the OVX group compared to the control group. Supplementation with probiotics increased VitD concentration in all combination groups, but this difference was only significant (*P* ≤ 0.05) in the combination groups 4,6 and 7 compared to both control and OVX groups (Fig. 3B).

The phosphorus concentration (P) was not significantly different between all experimental groups (Fig. 3C).

As the results showed, ALP concentration was significantly (*P* ≤ 0.05) more remarkable in the OVX group than in the control group. Treatment with combination groups 4, 6, and 7 considerably reduced ALP concentration. In other groups, ALP concentrations were elevated after treatment with probiotic combinations compared to the control group (Fig. 3D).

## Discussion

Bone is a pivotal system of the human body, and its hemostasis is related to intestinal flora because it is reported that the gut microbiome can considerably affect bone physiology [19, 20]. Maintaining the gut flora equilibrium is achieved by dietary changes or using probiotics and their metabolites (oligosaccharides, carbohydrates, fibers). Probiotics can change the gut microbiota composition, induce anti-inflammatory responses, endorse intestinal calcium absorption, and thus increase BMD [20].

Previous research has unequivocally shown that the intestinal system significantly impacts bone health. One way this happens is by controlling the absorption of minerals like calcium and phosphorus, which are essential for strong bones. Additionally, incretins and serotonin, which are obtained from the gut, and endocrine variables that affect the absorption of these minerals, might affect bone turnover [21, 22]. More recent research has shown how the intestinal microbiome affects bone function using germ-free rat and probiotics [23]. Rats treated with yogurt containing *L. casei, L. reuteri, and L. gasseri* enhanced calcium absorption, resulting in raised BMC relative to the control in research similar to the mouse model [24]. Similarly, supplementing developing rats with *L. rhamnosus* (HN001) enhanced calcium retention [25]. *B. longum* has been found to affect bone in addition to several Lactobacillus strains positively. Male rats supplemented with *B. longum* (ATCC 15,707) for 28 days had a higher percentage of fracture strength and more calcium and vitamin D in their tibias than untreated rats did [11]. In a different study, rats with a high-cholesterol diet plus fermented broccoli for 12 weeks showed a decrease in the number of TRAP-positive osteoclasts compared to untreated rats [26]. The probiotic combinations used in this study (groups 4, 6, and 7) that included *L. acidophilus, B. longum, L. reuteri, L. casei, and B. coagulans* significantly enhanced Vitamin D and Ca absorption.

The use of probiotic compounds is believed to have several potential advantages over single-strain formulations, including a greater chance of successful treatment by increasing probiotic strains, more potential niche, and a more comprehensive range of efficacy due to the greater diversity of strains, additive or synergistic effects due to increased adhesion, creation of a favorable environment and reduce intestinal microbiota antagonism [27]. However, the probiotic combination is not always successful and can sometimes have a potentially harmful effect due to the antagonistic effects between the probiotics in the product. Therefore, designing an experiment using multidimensional settings of multi-strain probiotics for such studies can be very useful [27]. Some preliminary studies reported that a VSL#3 (containing three species of *Bifidobacterium* (*B. longum, B. breve,* and *B. infantis*), four strains of *Lactobacillus* species (*L. casei, L. plantarum, L. acidophilus,* and *L. delbruekii subsp. bulgaricus*) has been used to treat osteoporosis [28, 29]. These *in-vivo* studies showed that this branded combination obviously improved the femoral bone density, trabecular thickness, and number [30]. This may be due to the spinal bone volume after ovariectomy in rats treated with VSL#3.

Although numerous investigations have studied the effect of a probiotic combination in the prevention and treatment of osteoporosis in animal models, there is much diversity among their strains. Since most of these compound strains are naturally present and formulated, there is no consensus on the most effective probiotic compound. Among the studies that examined the effect of probiotic combinations on the prevention and treatment of osteoporosis, we discussed in depth the familiar strains. Despite all the differences and similarities, our research team finally concluded that common strains found in the most effective probiotic combinations for osteoporosis are *Lactobacillus acidophilus, Lacticaseibacillus casei, Bifidobacterium longum, Bacillus coagulans, Limosilactobacillus reuteri.* Although this conclusion was not based on a systematic review study, it was based on the experiences of expert clinicians in the treatment of osteoporosis and manufacturers of probiotic formulations. In the next step, we divided these strains into several hybrid groups using a mathematical matrix, divided them into seven groups, and examined their preventive effects on osteoporosis in rat. Therefore, we inspected the effectiveness of seven probiotic combinations from five native probiotics *(Lactobacillus acidophilus, Limosilactobacillus reuteri, Lacticaseibacillus casei, Bifidobacterium longum,* and *Bacillus coagulans*), as mentioned in Table 1, on BMD, BMC, of global, spine, femur, and tibia on ovariectomized rats.

In this study, combination NO 4, containing *L. acidophilus, B. longum,* and *L. reuteri,* is the most influential group on global, spine, and femur BMD. Combination NO 3, containing *L. acidophilus*, *L. casei*, and *L. reuteri,* also significantly affects the BMD of the tibia among the treatment group. According to Kim et al., *L. casei* considerably improved the tibia BMD in OVX rats [31]. While evaluating the effects of our probiotic combinations on femur, spine, global, and tibia, we indicated that particular combination had the most influence on the special bone type. It was evident that combination NO 4 had the most significant increasing effect on global BMC. Also, combination NO 1 (comprising *L. acidophilus, L. casei,* and *B. longum)*, NO 6 (containing *L. casei, B. longum,* and *Bacillus coagulans),* NO 7 (containing *L. casei, L. reuteri,* and *B. longum),* and NO 4 had the most considerable raising effect on spine BMC.

What can be deduced from this study is that *L. acidophilus* is a common strain in the most effective combination sets tested to improve both BMC and BMC. Our previous study reported that *L. acidophilus* was more effective in the treatment groups in the case of global, spine, and femur BMD than the OVX untreated group [3]. Some studies reported that *L. reuteri* might decrease fracture, increase BMD, BMC, and trabecular number and thickness and weaken the trabecular space of the vertebrae and femurs. Inflammation may be the leading cause of abnormal bone regeneration and the onset of bone loss. Some studies have shown that increased inflammatory cytokines are associated with osteoclastic bone resorption, low bone mineral density (BMD), elevated bone resorption, and increased fracture risk. Therefore, blockade of the pro-inflammatory cytokine levels due to intake of probiotic supplements leads to a decrease in bone resorption [32, 33]. We also observed that this strain significantly improved global BMD, BMC, femur BMD, and BMC [3].

Also, *B. longum* is present in many effective combinations of this study, especially NO 4. Under our previous studies, *B. longum* could successfully affect femur BMD and BMC. The impact of *B. longum* on bone density, bone mineral content, bone remodeling, bone structure, and osteoclast/osteoblast gene expression markers was previously evaluated in the OVX rat model for 16 weeks and improved their bone density, trabecular number, thickness, and femoral strength. *B. longum* likewise reduces levels of serum C-terminal telopeptide [34].

The current study disclosed an apparent synergistic effect between *Lactobacillus* species and *B. longum*. Evidence studies in preclinical suggest that dual colonization of *Lactobacillus* sp. and *Bifidobacterium* sp. ameliorated severe diarrhea and synergistically decreased virus shedding titers probably because of modulating mucosal and systemic innate and adaptive immunity [35]. This synergistic effect may also be conceivable for the treatment and prevention of osteoporosis. Also, *Lactobacillus sp.* and *Bifidobacterium sp.* combination synergistically alleviated immobilization stress and anxiety behaviors, nuclear factor kappa-light-chain-enhancer of activated B cells (NF-κB) activation, brain-derived neurotrophic factor (BDNF) expression, tumor necrosis factor (TNF)-α, interleukin (IL)- 6, and lipopolysaccharide levels via maintaining of the gut immune responses and microbiota arrangement [36]. Additionally, these strains repressed the expression of NF-κB activation and TNF-α. Some recent studies indicated such an effect on bone mineralization when naturally combined *Lactobacillus* sp. and *Bifidobacterium* sp., attributed to changes in gut microbiota and ecology [37]. Most studies that have evaluated the effectiveness of probiotic compounds include *Lactobacillus* sp. In some studies, however, *Bifidobacterium* sp. was used in combination with *Lactobacillus*. This indicates the greater effectiveness of *lactobacilli* sp. in a mixture. *Bifidobacterium* sp. was used in most studies that did not show a more significant effect of the probiotic combinations, indicating that the therapeutic effect of *Bifidobacterium* sp. may be repressed when other species exist in a multivalent probiotic mixture. Among the studies in which the mixture was not more effective, all contained one or more species of *lactobacilli* along with *Bifidobacterium sp.* and several other genera. This reduction in effect indicates that sometimes the high diversity of strains in a probiotic combination diminishes the effectiveness of a multivariate probiotic. These diverse strains appear to constrain each other in the body environment, possibly due to the secretion of antagonistic agents or struggle for nutrients or receptors in the gastrointestinal tract. Our previous study revealed that oral administration of *B. longum* or *Lactobacillus* strains declines BMD and BMC criteria induced thorough ovariectomizing in response to hormone deficiency. However, the mixture of these probiotics was more effective in osteoporotic rats at all levels of BMD and BMC. This study’s results indicated that probiotic’s ameliorative effect on bone loss in ovariectomized rat model is strain-specific. The consequences have also revealed a discrepancy concerning the influence of these different probiotic treatments on releasing plasma levels of hormones. It seems that the *L. acidophilus* strain, combined with L. casei and B. longum strains, regulated immune systems, increased calcium absorption, and even ameliorated hormone levels under postmenopausal like osteoporosis conditions. The role of *B. longum* in the mixture of other strains can be elucidated by its anti-inflammatory features. The action of *B. longum* depends on the inflammatory state of the intestine, and it induces an anti-inflammatory response in inflammatory bowel disease. The anti-inflammatory properties of *B. longum* may be due to its capability to prevent the binding of pathogens to epithelial cells. Considering the functional specificity of each strain at the peripheral and central levels, all these data demonstrate that CNS function is improved by a mixture of these two or three probiotics (*L. Acidophilus*, *B. Longum,* and *L. casei*). This combination of probiotics is feasible and acceptable to people worldwide with no severe side effects. Although probiotic nutraceutical products have been extensively used for a long time, the use of probiotics to improve general health is increasing. For example, in a recent data network survey, approximately 40% of people indicated that in 2018 they used probiotics in various forms. Probiotic combinations are easy to process, formulate, commercialize, and prescribe in commercial conditions. Three effective probiotic strains in this study are available in various dairy products and other human fermented foods. They can be easily isolated and purified using conventional microbiological methods. Because of the growing interest in probiotics and their association with osteoporosis and bone loss, it is interesting to analyze the cost-effectiveness ratio of this potential intervention for osteoporosis.

However, the cost-effectiveness ratio of probiotic combinations for osteoporosis has not been estimated in the literature. Therefore, evaluating the cost-effectiveness of using probiotic combinations to prevent osteoporosis in postmenopausal women can lead to valuable results.

The limitation of our study was that we did not detect the changes of related metabolites of intestinal flora, metabolic indexes of bacterial flora and bone related pathway proteins, bone remodeling related indexes, and bone microstructure.

## Conclusion

Our observations revealed that probiotic combinations could be used for bone formation improvement, bone resorption reduction, and microstructure of femur changing. Among seven combined effective probiotics, a combination containing *L. acidophilus, B. longum,* and *L. reuteri* is the most influential group in ovariectomized osteoporotic rat. This combination synergistically increased the level of BMC and BMD together with calcium absorption, osteoblast activity, and vitamin D concentration that cause elevated bone health. Further clinical studies are needed to ensure that these reports are qualified for human beings.

## Data Availability

The datasets generated during and/or analyzed during the current study are available from the corresponding author on reasonable request.
